# Sources and pathways of carbon and nitrogen of macrophytes and sediments using stable isotopes in Al-Kharrar Lagoon, eastern Red Sea coast, Saudi Arabia

**DOI:** 10.1371/journal.pone.0299562

**Published:** 2024-04-25

**Authors:** Ramadan H. Abu-Zied, Mohammed I. Orif, Rashad A. Bantan, Radwan Al-Farawati, Mohammed A. Ghandourah, Mohammed H. Aljahdali

**Affiliations:** 1 Marine Geology Department, Faculty of Marine Sciences, King Abdulaziz University, Jeddah, Saudi Arabia; 2 Geology Department, Faculty of Science, Mansoura University, El-Mansoura, Egypt; 3 Marine Chemistry Department, Faculty of Marine Sciences, King Abdulaziz University, Jeddah, Saudi Arabia; King Saud University, SAUDI ARABIA

## Abstract

Elemental ratios (δ^13^C, δ^15^N and C/N) and carbon and nitrogen concentrations in macrophytes, sediments and sponges of the hypersaline Al-Kharrar Lagoon (KL), central eastern Red Sea coast, were measured to distinguish their sources, pathways and see how they have been influenced by biogeochemical processes and terrestrial inputs. The mangroves and halophytes showed the most depleted δ^13^C values of –27.07±0.2 ‰ and –28.34±0.4 ‰, respectively, indicating their preferential ^12^C uptake, similar to C3-photosynthetic plants, except for the halophytes *Atriplex* sp. and *Suaeda vermiculata* which showed δ^13^C of –14.31±0.6 ‰, similar to C4-plants. Macroalgae were divided into A and B groups based on their δ^13^C values. The δ^13^C of macroalgae A averaged –15.41±0.4 ‰, whereas macroalgae B and seagrasses showed values of –7.41±0.8 ‰ and –7.98 ‰, suggesting uptake of HCO_3_^–^ as a source for CO_2_ during photosynthesis. The δ^13^C of sponges was –10.7±0.3 ‰, suggesting that macroalgae and seagrasses are their main favoured diets. Substrates of all these taxa showed δ^13^C of –15.52±0.8 ‰, suggesting the KL is at present a macroalgae-dominated lagoon. The δ^15^N in taxa/sediments averaged 1.68 ‰, suggesting that atmospheric N_2_-fixation is the main source of nitrogen in/around the lagoon. The heaviest δ^15^N (10.58 ‰) in halophytes growing in algal mats and sabkha is possibly due to denitrification and ammonia evaporation. The macrophytes in the KL showed high C %, N %, and C/N ratios, but this is not indicated in their substrates due possibly to a rapid turnover of dense, hypersaline waters carrying most of the detached organic materials out into the Red Sea. The δ^13^C allowed separation of subaerial from aquatic macrophytes, a proxy that could be used when interpreting paleo-sea level or paleoclimatic changes from the coastal marine sediments.

## Introduction

The coastal lagoons of the Red Sea and world gain a special attention due to their importance for the adjacent marine waters as they provide them with organic carbon and nutrients derived from decomposition of primary producers such as mangroves, halophytes, seagrasses, and macroalgae [1, 2]. Al-Kharrar Lagoon is located at the centre of the eastern Red Sea coast and is connected to the most oligotrophic basin in the world [3, 4].

In order to know organic matter sources/pathways, nutrient cycling and storage in coastal systems, and to help determine an organism’s trophic position, the carbon and nitrogen stable isotopes have been employed extensively on their macrophyte tissues and substrate sediments. For example, the C/N ratio and δ^15^N of seagrasses were used as a source indicator of dissolved inorganic nitrogen (DIN) in reef environments, where the δ^15^N could have undergone changes due to high concentrations of nitrogen that allowed more fractionation or an increased uptake of land-derived DIN with high δ^15^N value [5]. On the other hand, the δ^13^C reflects source carbon, irradiance, temperature and becomes more enriched when vigorous photosynthesis occurs [6, 7]. In a greenhouse-based simulated mangrove wetland, increasing salinity and water levels significantly increased the δ^13^C and δ^15^N values in plant organs, but after treatment with salinity of more than 30 ‰, the δ^15^N value of plant organs did not increase implying that changes in salinity and water levels due to climatic changes may impact N cycling processes in wetland systems [8].

The δ^13^C in two mangrove stands and along the Tanzanian coast ranged from –25.9 to –29.1 ‰ suggesting that mangrove trees in the two stands follow the C3-type of photosynthetic pathway, whereas the δ^15^N ranged from –1.5 to 3.2 ‰ indicating atmospheric nitrogen fixation by mangrove plants. However, these isotopic signatures were not indicated in the sediments beneath the mangroves that showed low C/N ratios and low enrichment in ^13^C and ^15^N relative to the plant material owing to mixing of nitrogenous-rich material from an adjacent area [9]. A study employed the δ^13^C and δ^15^N of algae, seagrasses and particulate organic matter (POM) in Biscayne Bay (USA) to differentiate between natural and anthropogenic inputs and concluded that the main source of ^15^N enrichment (7–10 ‰) close to the coast (<1 km) is from sewage influenced waste water rather than regeneration, and this enrichment diminishes quickly with increasing distance from the coast [10]. It also suggested that δ^15^N values >8 ‰ can be used as a tracer of anthropogenic input in certain geographical locations. A similar study was carried out on the nitrogen sources around the British isles using the δ^15^N of the seagrass meadows, where values of δ^15^N ranged from 3.15 to 20.16 ‰ (mean of 8.69 ‰), and were high within the Thames Basin suggesting a significant influx of urban sewage and livestock effluent into the system [11]. These findings are consistent with another study [12] where more positive values are thought to be indicative of anthropogenic nitrogen input, while values closer to zero are considered typical of more pristine systems. However, it ascribed enriched δ^15^N values not only to anthropogenic input but they could be also related to natural processes removing N from the environment, leaving the pool enriched in ^15^N and hence leading to enriched δ^15^N in organisms [12].

The C/N ratio, δ^13^C and δ^15^N values of surface sediment organic matter (SOM) of marine coastal waters were used by many authors [13–15] as tools to differentiate between sources of organic matter (OM) in sediments whether from marine or terrestrial or anthropogenic sources. It is reported that a C/N ratio greater than 15 indicates a terrestrial origin and ratio of 4–10 indicates a marine source, whereas a value of 10–15 indicates components of both marine and terrestrial origins in the sediment source [16, 17].

The carbon and nitrogen stable isotopes of the Red Sea are not well explored, where few studies have been carried out on the δ^13^C and δ^15^N of macrophytes throughout the coastal marine environments of the Red Sea [4, 15, 18] as well as on the zooplankton, phytoplankton and POM of open marine waters [3]. These studies concluded that the δ^15^N values of both coastal and open marine waters decrease towards the north following the salinity gradient increase, indicating profuse atmospheric N_2_-fixation in the northern Red Sea, whereas to the south the δ^15^N increases due to a dominance of NO_3_^‒^ coming from the Indian Ocean waters. Also, N concentrations and stocks decreased from south to north in seagrass sediments, matching the productivity gradient, while organic carbon concentrations and stocks were uniform [18]. It is also noted that the aquatic mangrove habitats in the Red Sea are characterized by having a low sink of organic carbon possibly due to the occurrence of extreme environmental conditions, such as low rainfall, nutrient limitation and high temperature, reducing the growth rates of the mangroves and increasing soil respiration rates [15].

**Fig 1:**
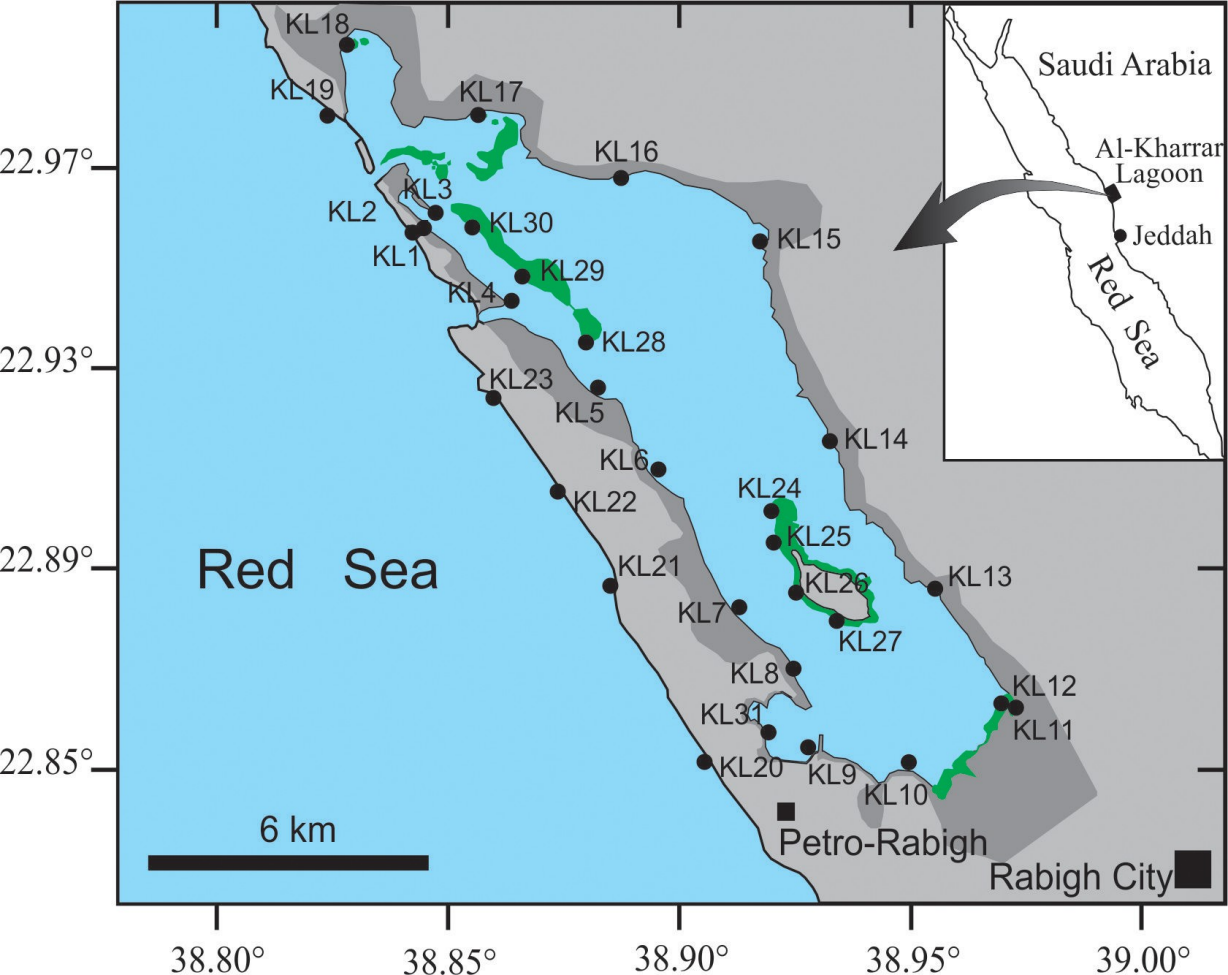
Location map of Al-Kharrar Lagoon showing the station sites (solid black circle). Dense mangrove areas indicated by green colour and supratidal areas are indicated by dark grey colour. The map outline was taken from the national geological database portal licensed to the Saudi Geological Survey (https://ngp.sgs.gov.sa/).

Al-Kharrar Lagoon (KL) is one of the Red Sea lagoonal systems, extending in an elongate shape for about 20 km parallel to the Red Sea coast (Fig 1). The KL is located under dry, warm, tropical climate (Fig 1). Its sediment archives indicate a sensitivity of the lagoon to late Holocene climate variability [19]. Thus, the study of δ^13^C, δ^15^N, C/N and percentages of C and N in the present lagoon’s macrophytes may provide useful information to understand the Holocene climatic variability in the Red Sea region. The KL is connected with the Red Sea via a narrow (120 m wide) and deep (14 m) opening at its northwestern side [20, 21]. This restricts its water renewal with the Red Sea making it a hypersaline basin with average water salinity and temperature of 41 ‰ and 31°C, respectively, showing a north-south gradient [20, 21]. The southern part of the KL is connected with an intermittent wadi (Wadi Rabigh) which no longer reaches the lagoon due to the 2009 construction of Rabigh Dam about 35 km east of Rabigh City [21]. These conditions make the lagoon prosperous with a fauna and flora of mangroves, halophytes, seagrasses, macroalgae, corals, as well as fishes and sea turtles which use the lagoon as a nursery for their youngsters. These organisms, after death, release essential nutrients into the adjacent oligotrophic Red Sea waters sustaining many food-web systems.

This study aims to measure δ^13^C, δ^15^N, C/N and % C and % N in macrophytes (mangrove *Avicennia marina*, halophytes, macroalgae and seagrasses), sponges and sediment substrates of the KL and adjacent shallow waters of the Red Sea in order to distinguish C and N sources and pathways in the lagoon and how much they have been influenced by geochemical processes and terrestrial inputs. This study also hypothesized that (1) whether the δ^15^N of all macrophytes are the same, and close to the atmospheric nitrogen or different; and (2) the δ^13^C values of the lagoonal mangroves, macroalgae and seagrasses are nearly similar to those of the adjacent shallow waters of the Red Sea. Since this lagoon is at present in a natural condition far from urban activities, the results of this study could be used as a natural reference when assessing human-impacted lagoons, having a similar geographic setting.

## Materials and methods

Thirty one stations in/around the Al-Kharrar Lagoon (KL) were surveyed for their macrophytes, substrate sediments and sponges during October 2020 (Fig 1). A permission was obtained from the Saudi Coast Guard of Makkah region (Ministry of Interior) prior visiting these sites. At these stations, a total of 176 samples were collected from mangroves *Avicennia marina* (42), halophytes (28), macroalgae (48), seagrasses (22), sponges (7) and sediments (29). The station’s coordinates, description and their represented samples are displayed in Table 1. Mangrove leaves and aerial roots (pneumatophores) and halophyte leaves were collected by hand with a knife as follows: mangroves from the intertidal area; and halophytes from supratidal areas enriched in algal mats as well as from raised, sandy beaches. During snorkelling, macroalgae and seagrasses (leaves and rhizomes) were collected by hand with a knife from the intertidal and subtidal areas at water depths ranging from 0.5 to 1 m. The sponges were collected with a knife wherever they were found. Substrate sediment samples were collected at each station with a stainless steel scoop shovel from underneath mangrove trees, halophytes (growing in algal mats) and seagrasses and from vegetation-free substrates.

**Table 1:** A list of the surveyed stations and their coordinates and the representative samples of macrophytes (mangroves, halophytes, macroalgae and seagrasses), sponges and sediments at each station. Letter P indicates the presence of taxa and sediments samples.

S. No	Latitude	Longitude	Mangroves		Halophytes					Macroalgae													Seagrasses						Sponges			Sediments
			*A*. *marina* (leaves)	*A*. *marina* (roots)	*Anabasis setifera*	*Atriplex* sp.	*Salicornia fruticosa*	*Suaeda vermiculata*	*Zygophy coccineum*	*Acanthophora* sp.	*Caulerpa racemosa*	*Codium* cf. *C*. *edule*	*Colpomenia sinuosa*	Cyanobacteria	*Digenea simplex*	*Digenea* sp.	*Laurencia obtusa*	*Padina boryana*	*Sargassum latifolium*	*Sargassum* sp. 1	*Sargassum* sp. 2	*Turbinaria conoids*	*C*. *rotundata*, leaves	*H*. *stipulacea*, leaves	*T*. *hemprichii*, leaves	*C*. *rotundata*, roots	*H*. *stipulacea*, roots	*T*. *hemprichii*, roots	*Hyrtios erectus*	*Demospongiae* sp.	Sponge (unknown)	
KL1	22.957052°	38.842416°	P	P			P			P				P																		P
KL2	22.957660°	38.844350°																														P
KL3	22.959954°	38.846851°	P	P													P							p								P
KL4	22.943214°	38.863484°	P	P			P																	P								P
KL5	22.925980°	38.882169°	P	P			P			P			P							P		P										P
KL6	22.909654°	38.895413°	P	P	P					P			P							P		P		P			P					P
KL7	22.881830°	38.912753°	P	P			P			P		P			P		P		P			P		P							P	P
KL8	22.869933°	38.924575°					P													P				P							P	P
KL9	22.854456°	38.927565°			P		P													P			P							P		
KL10	22.851010°	38.949204°			P		P			P			P											P								P
KL11	22.861575°	38.972485°																														P
KL12	22.862384°	38.969398°	P	P										P			P							P								P
KL13	22.885887°	38.955178°	P	P																				P								P
KL14	22.915115°	38.932422°	P	P	P				P																							P
KL15	22.955133°	38.917260°	P	P																			P	P								P
KL16	22.967929°	38.887286°							P																							
KL17	22.980615°	38.856367°	P	P							P																					P
KL18	22.994418°	38.827968°	P	P																												P
KL19	22.980550°	38.824317°	P	P	P				P	P												P										P
KL20	22.851779°	38.905321°				P		P	P						P		P													P		P
KL21	22.886383°	38.885163°				P		P	P	P															P			P				P
KL22	22.905523°	38.873889°				P		P	P								P					P			P			P				P
KL23	22.923982°	38.859576°				P			P								P								P			P				P
KL24	22.901290°	38.919646°	P	P						P				P									P			P						P
KL25	22.895182°	38.920185°	P	P													P				P											P
KL26	22.884836°	38.924849°	P	P						P							P	P					P						P			P
KL27	22.879264°	38.933989°	P	P						P																						P
KL28	22.935020°	38.879564°	P	P										P			P			P										P	P	P
KL29	22.947876°	38.865848°	P	P												P	P															P
KL30	22.957711°	38.855001°													P																	
KL31	22.856632°	38.919671°			P		P			P										P				P								P

During field collection, the mangroves, macroalgae and seagrasses samples were, in situ, rinsed in seawater to remove adhered mud, then all samples were inserted in zip-lock plastic bags and closed tightly. Immediately, all samples were transferred to a portable ice box until they reached the laboratory on the same day, where all samples were kept in a fridge below 5°C. In the laboratory (next day), the macrophytes and sponges were sorted and identified into genus/species level. Acidification of macroalgae, seagrasses and aerial roots of mangroves with 1M HCl to remove epiphytes and adhered carbonates was followed, because it is recommended by many authors [13, 18], giving a reasonable and unbiased results for δ^13^C and δ^15^N analyses in organic matters. Hereafter, all samples of the identified taxa to be processed for isotopic analysis were washed three times with ultrapure (milli-Q) waters. Carbonates in the sediment samples (approximately 1 g dry wt) were also removed by 1M HCl solution before washing, centrifuging and decantation 3 times with ultrapure waters. After that, all processed taxa and sediments were oven-dried at 60°C until constant weight was reached. Each dried sample was inserted into a zip-lock plastic bag and closed tightly, then all plastic bags with dried samples were stored in desiccators to maintain complete dryness until running the isotopic analyses.

To measure the elemental δ^13^C, δ^15^N and C/N and percentages of C and N in the dried macrophyte taxa, sediment and sponge samples, approximately one leaf and 1 cm-thick piece of root/rhizome of each mangrove *Avicennia marina* and seagrass taxa, 1–2 leaves of each macroalgae and halophyte taxa, 0.5 cm^3^ of each sponge and whole acidified sediment samples were homogenized into powder using an agate mortar and pestle. The mortar and pestle were carefully cleaned after grinding each sample to insure no contamination transferred to the next sample. Then immediately a weighed-portion (approximately 5–8 mg for mangroves and halophytes, 3–5 mg for macroalgae and seagrasses, 0.2–0.3 mg for sponges and 7–10 mg for sediments) from the powdered samples were inserted in tin boats, wrapped and inserted in the autosampler (80 holes) of the Elementar varioISOTOPE cube elemental analyser (EA) connected to an Isoprime100 IRMS (UK) at the isotope laboratory, Faculty of Marine Sciences, King Abdulaziz University, Jeddah. The samples were combusted in the EA at 950°C (CN mode), then purged in a continuous He flow to the IRMS.

The isotopic composition of samples were expressed as delta value (δ) and calculated relatively to PeeDee Belemnite (PDB) standard for carbon and atmospheric N_2_ for nitrogen using the following equation δ ‰ = (R_Sample_/R_Standard_− 1) * 1000 (units per mil, ‰), where R = ^13^C/^12^C for δ^13^C values and ^15^N/^14^N for δ^15^N values. The laboratory working (reference) gases CO_2_ and N_2_ were calibrated against the IAEA reference materials: IAEA-N-1, IAEA-N-2 for δ^15^N and IAEA-CH-3, IAEA-CH-6, IAEA-CH-7 for δ^13^C. During the analysis, we also used two internal standards (STD) inserted systematically in batches every 10 unknown samples. The first STD is acetanilide #1 with δ^13^C = –27.75 ‰ and δ^15^N = 1.61 ‰ and the second STD is an inter-laboratory standard EEZ23 given by the Isotope Laboratory of CSIC-UGR, Spain with δ^13^C = –13.25 ‰ and δ^15^N = 16.01 ‰. The analytical precision (standard deviation) of these standards was better than 0.05 ‰ for δ^13^C and 0.2 ‰ for δ^15^N. The reproducibility of these standards was used to check the stability, linearity and daily factors of the EA-IRMS. A complete list of the mean (±SE, standard error) values of C % and N %, C/N ratio, δ^13^C and δ^15^N of studied macrophytes, sediments and sponges (176 analysed samples) are provided in the supporting information (S1 Table). The obtained values of these parameters are comparable with those recorded for the same macrophytes in the Red Sea [4, 15, 18] and worldwide [12, 22, 23].

Ordinary least squares regression and correlation of the total data (C %, N %, δ^13^C, δ^15^N and C/N ratio) were obtained using linear bivariate model of PAST software, version 4.04 [24]. In this model, the bootstrapped confidence interval was 95% and significance level (*p-value*) equals 0.05. Other statistical techniques such as cluster and PCA, or hypothesis testing by ANOVA did not show any significant results.

## Results

### Mangroves

**Fig 2:**
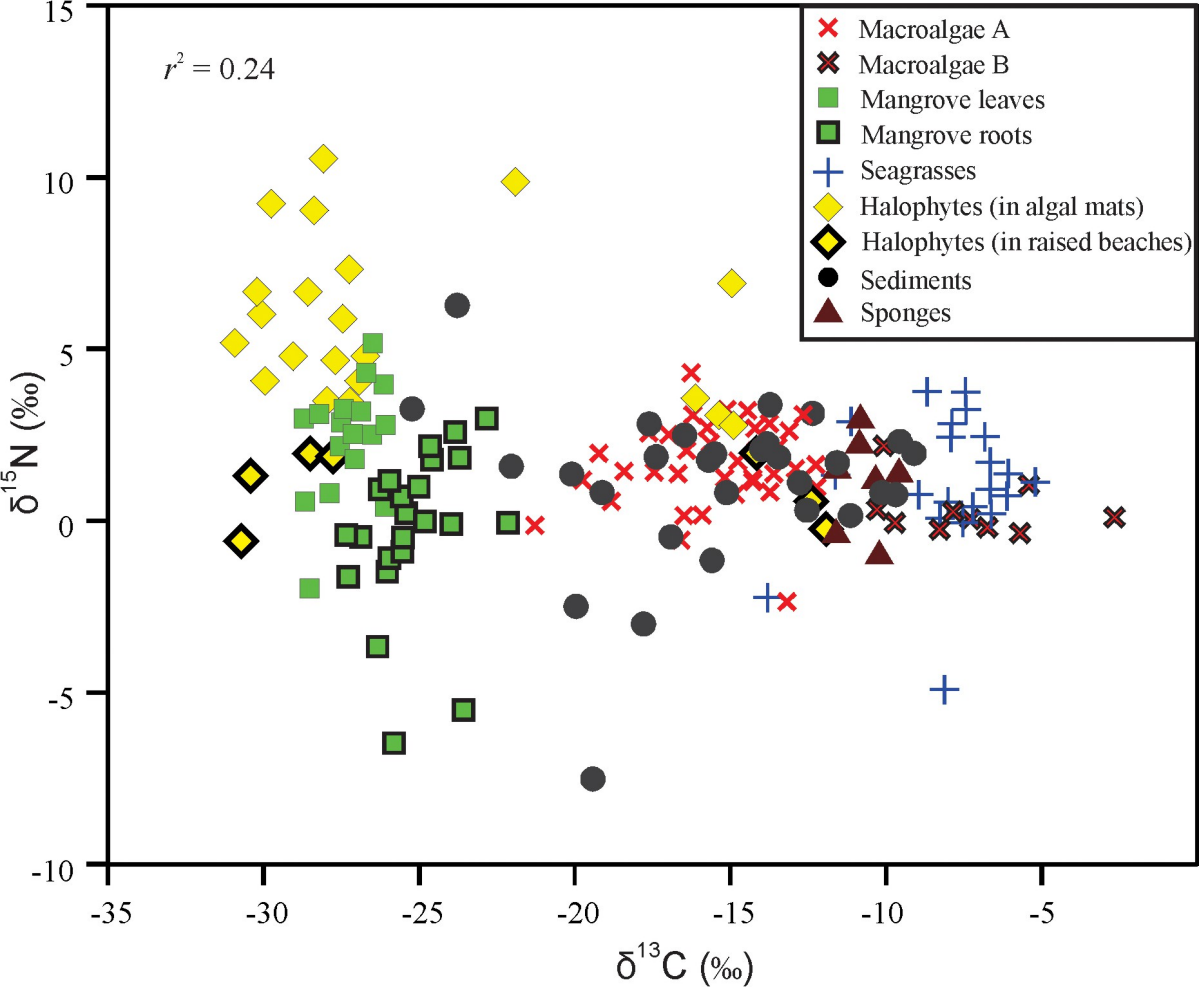
Relationship between δ^13^C and δ^15^N of the macrophytes (mangroves, halophytes, macroalgae and seagrasses), sediments and sponges in/around the Al-Kharrar Lagoon.

**Fig 3:**
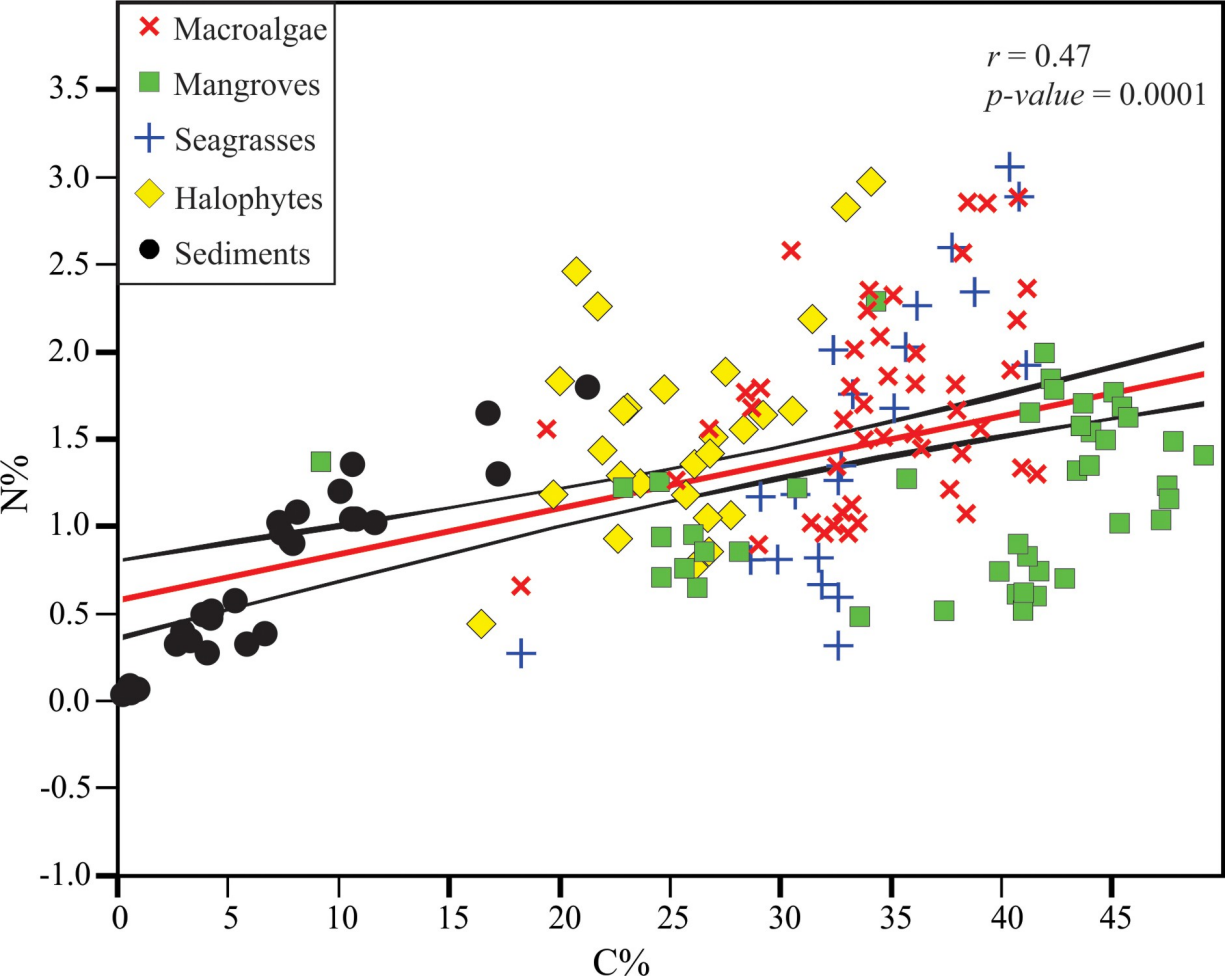
Relationship between C % and N % of the macrophytes (mangroves, halophytes, macroalgae and seagrasses) and sediments in/around the Al-Kharrar Lagoon. Red solid line indicates the fitted regression line between all macrophytes and sediments. The 95% confidence interval is indicated by black solid line.

**Fig 4:**
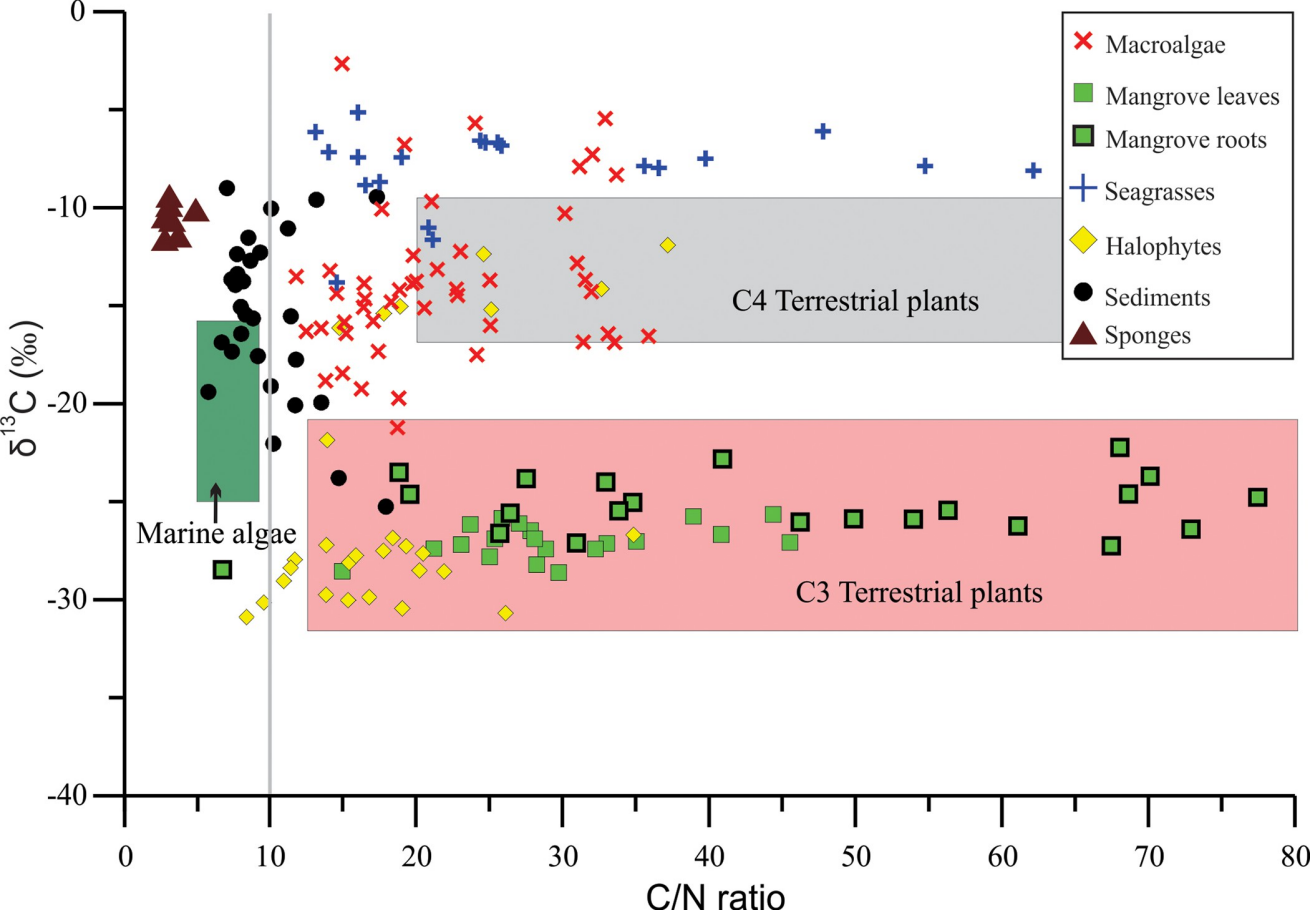
Relationship between C/N ratio and δ^13^C of the macrophytes (mangroves, halophytes, macroalgae and seagrasses), sediments and sponges in/around the Al-Kharrar Lagoon. The vertical grey line is placed at C/N ratio = 10, a value separating the marine-influenced sediments from those of mostly terrestrial setting, following [16, 17]. Square/area plots of different taxa were taken from [13].

The δ^13^C of mangrove *Avicennia marina* leaves was very light with a mean (±SE) of –27.07±0.2 ‰ and ranged from –28.62 to –25.76 ‰, whereas the δ^13^C of their aerial roots (pneumatophores) increased slightly with a mean of –25.28±0.3 ‰ and ranged from –28.47 to –22.23 ‰, and with a difference from leaves of about 1.8 ‰ (Fig 2, Table 2). The δ^15^N of leaves had a mean of 1.81±0.6 ‰ (–6.46 to 5.06 ‰), and in the roots they in contrary decreased to a mean of 0.11±0.4 ‰, ranging from –5.49 to 3.00 ‰ (Fig 2, Table 2). The carbon (C %) and nitrogen (N %) concentrations and their C/N ratio in the mangrove leaves showed mean values of 43.83±0.9%, 1.57±0.1% and 29.21±1.7, whereas in the aerial roots they were 32.88±1.9%, 0.82±0.1% and 44.98±4.2, respectively (Fig 3, Table 2). It is noted that the aerial roots of mangroves showed the highest C/N ratios of 44.98±4.2 and ranged from 6.80 to 77.46, whereas their leaves decreased to 29.21±1.7 and ranged from 14.98 to 45.52 (Fig 4 and Table 2).

**Table 2:** Mean (±SE) and Min-Max values of C %, N %, C/N ratio, δ^13^C and δ^15^N in the macrophytes, sponges and sediments of the Al-Kharrar Lagoon. Count of samples indicated between two brackets. The macroalgae A includes species such as *Acanthophora* sp., *C*. cf. *C*. *edule*, green filamentous algae, *D*. *simplex*, *Digenea* sp., *L obtusa*, *S*. *latifolium*, *Sargassum* sp. 1 and *Sargassum* sp. 2. The macroalgae B includes species such as *C*. *racemosa*, *C*. *sinuosa*, *P*. *boryana* and *T*. *conoides*.

Taxa	C%		N%		C/N ratio		δ^13^C		δ^15^N	
	Mean	Min-Max	Mean	Min-Max	Mean	Min-Max	Mean	Min-Max	Mean	Min-Max
Mangroves:										
Leaves (19)	43.83±0.9	34.32–49.14	1.57±0.1	1.04–2.29	29.21±1.7	14.98–45.52	-27.07±0.2	-28.62 to -25.76	1.81±0.6	-6.46 to 5.06
Aerial roots (23)	32.88±1.9	9.08–45.34	0.82±0.1	0.48–1.34	44.98±4.2	6.80–77.46	-25.28±0.3	-28.47 to -22.23	0.11±0.4	-5.49 to 3.00
Halophytes:										
C3-species (21)	25.47±0.9	19.8–34.06	1.68±0.1	0.78–2.97	16.93±1.3	8.43–34.85	-28.34±0.4	-30.89 to -21.86	5.25±0.6	-0.55 to 10.58
C4-*Atriplex* sp. and *S*. *vermiculata* (7)	25.15±1.6	16.49–29.23	1.17±0.2	0.44–1.89	24.45±3.1	14.79–37.19	-14.31±0.6	-16.14 to -11.91	2.62±0.9	-0.34 to 6.90
Macroalgae:										
Macroalgae A (36)	34.59±0.8	8.56–41.45	1.79±0.1	0.96–2.88	20.46±1.2	3.79–35.87	-15.41±0.4	-21.23 to -12.23	1.60±0.2	-2.38 to 4.25
Macroalgae B (9)	32.45±2.1	18.26–41.16	1.21±0.2	0.66–2.34	27.72±1.7	17.67–33.69	-7.41±0.8	-10.29 to -2.66	0.33±0.3	-0.35 to 2.13
Seagrasses:										
Leaves (16)	34.70±1.0	28.78–41.06	1.78±0.2	0.59–3.07	23.18±2.7	13.14–54.75	-8.20±0.6	-13.81 to -5.14	1.71±0.4	-2.17 to 3.74
Rhizomes (6)	28.65±3.5	18.30–32.65	0.76±0.2	0.29–1.28	43.82±7.6	25.57–62.15	-7.30±0.4	-8.12 to -6.10	-0.70±1.1	-4.92 to 0.88
Sponges (7)	37.66±0.8	35.66–41.51	11.11±0.8	7.77–13.13	3.52±0.3	3.11–4.90	-10.72±0.3	-11.75 to -9.62	0.80±0.5	-1.02 to 2.23
Sediments (29)	6.84±1.0	0.25–21.27	0.70±0.1	0.04–1.81	9.95±0.6	5.83–17.95	-15.52±0.8	-25.25 to -9.00	1.11±0.5	-7.59 to 6.32

### Halophytes

The δ^13^C of halophytes divided them into depleted and enriched halophytes. The depleted δ^13^C halophytes (such as *Anabasis setifera*, *Salicornia fruticosa*, and *Zygophyllumm coccineum*) had a mean (±SE) of –28.34±0.4 ‰, and ranged from –30.89 to –21.86 ‰. Whereas, the enriched δ^13^C halophytes (such as *Atriplex* sp. and *Suaeda vermiculata*) had a mean of –14.31±0.6 ‰, and ranged from –16.14 to –11.91 ‰ (Fig 2, Table 2). The δ^15^N of halophytes had a mean of 4.59±0.6 ‰, and ranged from –0.55 to 10.58 ‰. The halophytes that grow in algal mats substrates showed more enriched δ^15^N values than those growing in raised, aerated sandy substrates (Fig 2). The C %, N % and their C/N ratio in the halophyte tissues showed mean values of 25.39±0.8%, 1.56±0.1% and 18.81±1.4, respectively (Figs 3 and 4 and Table 2). The halophyte tissues showed the lowest C/N ratio in the studied macrophytes.

### Macroalgae

The studied macroalgae in the Al-Kharrar Lagoon include the following: *Acanthophora* sp., *Caulerpa racemosa*, *Codium* cf. *C*. *edule*, *Colpomenia sinuosa*, green filamentous algae, *Digenea simplex*, *Digenea* sp., *Laurencia obtusa*, *Padina boryana*, *Sargassum latifolium*, *Sargassum* sp. 1, *Sargassum* sp. 2 and *Turbinaria conoides*. The δ^13^C values of macroalgae divided them into depleted-^13^C macroalgae A (*Acanthophora* sp., *C*. cf. *C*. *edule*, green filamentous algae, *D*. *simplex*, *Digenea* sp., *L obtusa*, *S*. *latifolium*, *Sargassum* sp. 1 and 2) and enriched-^13^C macroalgae B (*C*. *racemosa*, *C*. *sinuosa*, *P*. *boryana* and *T*. *conoides*) (Table 2). The depleted-^13^C macroalgae A consist mostly of red to brown algae, whereas the enriched-^13^C macroalgae B consists mostly of brown algae but with inclusion of green coloration. This division is in agreement with that reported by many authors [25, 26] that macroalgae with values <–30 ‰ tend to be red, whereas green macroalgae tend to have δ^13^C values >–10 ‰.

The δ^13^C of macroalgae A was the lighter with a mean of –15.41±0.4 ‰ and ranged from –21.23 to –12.23 ‰, whereas the δ^13^C of macroalgae B increased to a mean of –7.41±0.8 ‰ and ranged from –10.29 to –2.66 ‰ (Fig 2 and Table 2). Their δ^15^N values showed mean values of 1.60±0.2 ‰ (–2.38 to 4.25 ‰) and 0.33±0.3 ‰ (–0.35 to 2.13 ‰), respectively (Fig 2 and Table 2). The C %, N % and their C/N ratio in the macroalgae tissues showed mean value of 34.16±0.8%, 1.68±0.1% and 22.39±1, respectively (Figs 3 and 4 and Table 2). It is noted that the C/N ratio of macroalgae B with green coloration was higher (27.72±1.7) than the brown-red macroalgae A that have a mean of 20.46±1.2 (Table 2).

### Seagrasses

The seagrasses include the following species: *Cymodocea rotundata*, *Halophila stipulacea* and *Thalassia hemprichii*. Their δ^13^C values had mean of –7.98±0.4 ‰ and ranged from –13.81 to –5.14 ‰. The δ^13^C in seagrass rhizomes was slightly heavier (–7.30±0.4 ‰) than in their leaves (–8.20±0.6 ‰) by 0.9 ‰ (Table 2). The δ^15^N of leaves had a mean of 1.71±0.4 ‰ (–2.17 to 3.74 ‰), and in the rhizomes they decreased (as in the mangroves) to a mean of –0.70±1.1 ‰, ranging from –4.92 to 0.88 ‰ (Fig 2 and Table 2). The C % and N % and their C/N ratios in the seagrasses showed mean values of 33.49±1.2%, 1.58±0.2% and 27.3±3.2, respectively (Figs 3 and 4 and Table 2). It is noted that the rhizome of seagrasses showed the highest C/N ratios (as in mangrove roots) of 43.8±7.6 and ranged from 25.57 to 62.15, whereas their leaves decreased to 23.18±2.7 and ranged from 13.14 to 54.75 (Table 2).

### Sponges

Three genera of sponges were sampled such as *Demospongiae* sp., *Hyrtios erectus* and an unknown sponge. Their δ^13^C showed a mean of –10.72±0.3 ‰ and ranged from –11.75 to –9.62 ‰, whereas their δ^15^N showed a mean of 0.80±0.5 ‰ and ranged from –1.02 to 2.23 ‰ (Fig 2 and Table 2). The C % and N % and their C/N ratio in the sponges showed mean values of 37.66±0.8%, 11.11±0.8% and 3.52±0.3, respectively (Figs 3 and 4 and Table 2). The sponges showed a very low C/N ratio, because they are animals rich in N.

### Sediments

The δ^13^C of substrate sediment organic matter (SOM) showed a mean of –15.52±0.8 ‰, and ranged from –25.25 to –9.00 ‰, whereas their δ^15^N averaged ~1.11±0.5 ‰ and ranged from –7.59 to 6.32 ‰ (Fig 2 and Table 2). The C % and N % and their C/N ratio in the SOM showed mean values of 6.84±1.0%, 0.70±0.1% and 9.95±0.6, respectively (Figs 3, 4 and Table 2).

### Inter-relationships and correlation

**Fig 5:**
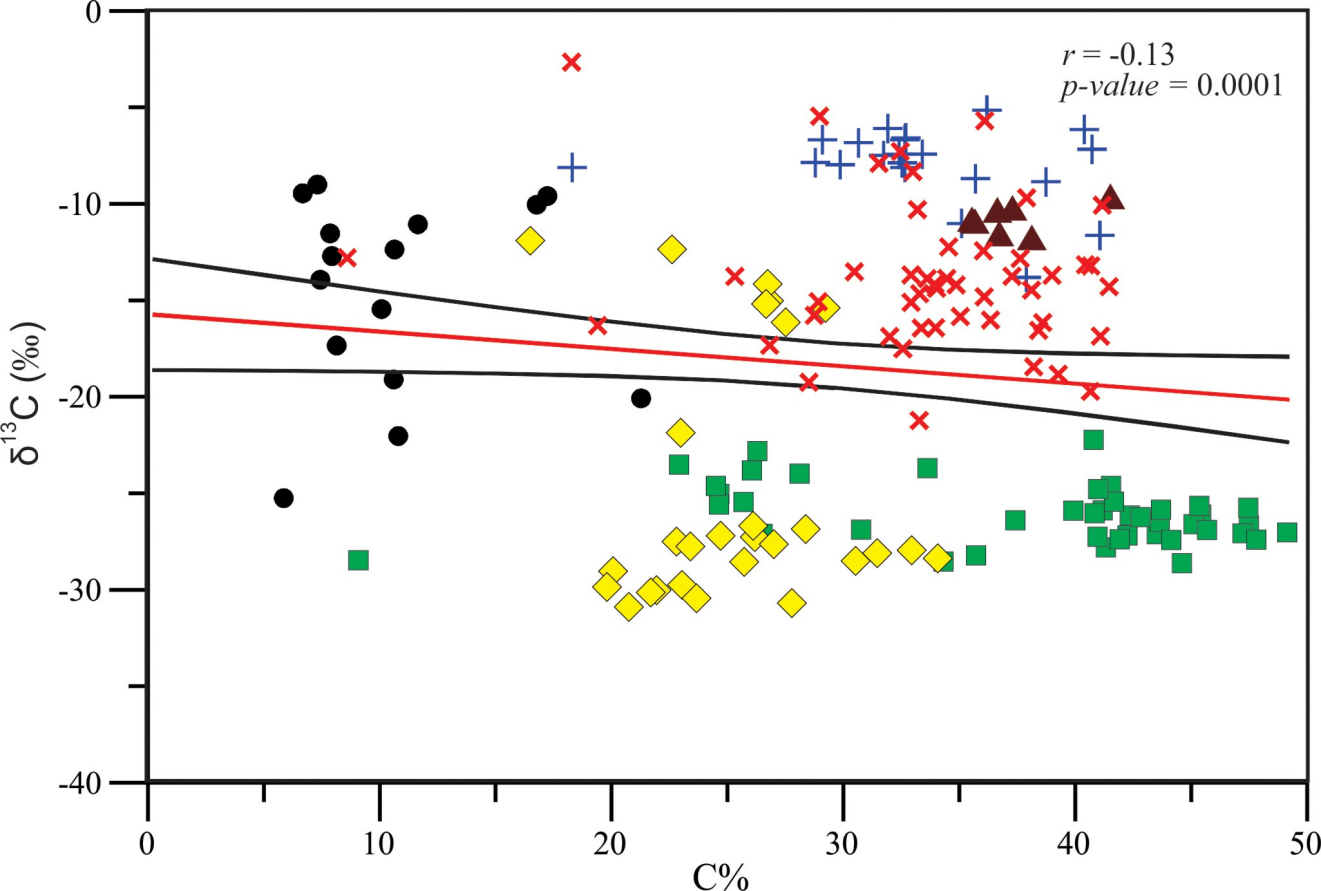
Relationship between C % and δ^13^C of the macrophytes (mangroves, halophytes, macroalgae and seagrasses), sediments and sponges in/around the Al-Kharrar Lagoon. Red solid line indicates the fitted regression line between all studied samples. The 95% confidence interval is indicated by black solid lines. For samples symbols see Figs 2–4.

**Fig 6:**
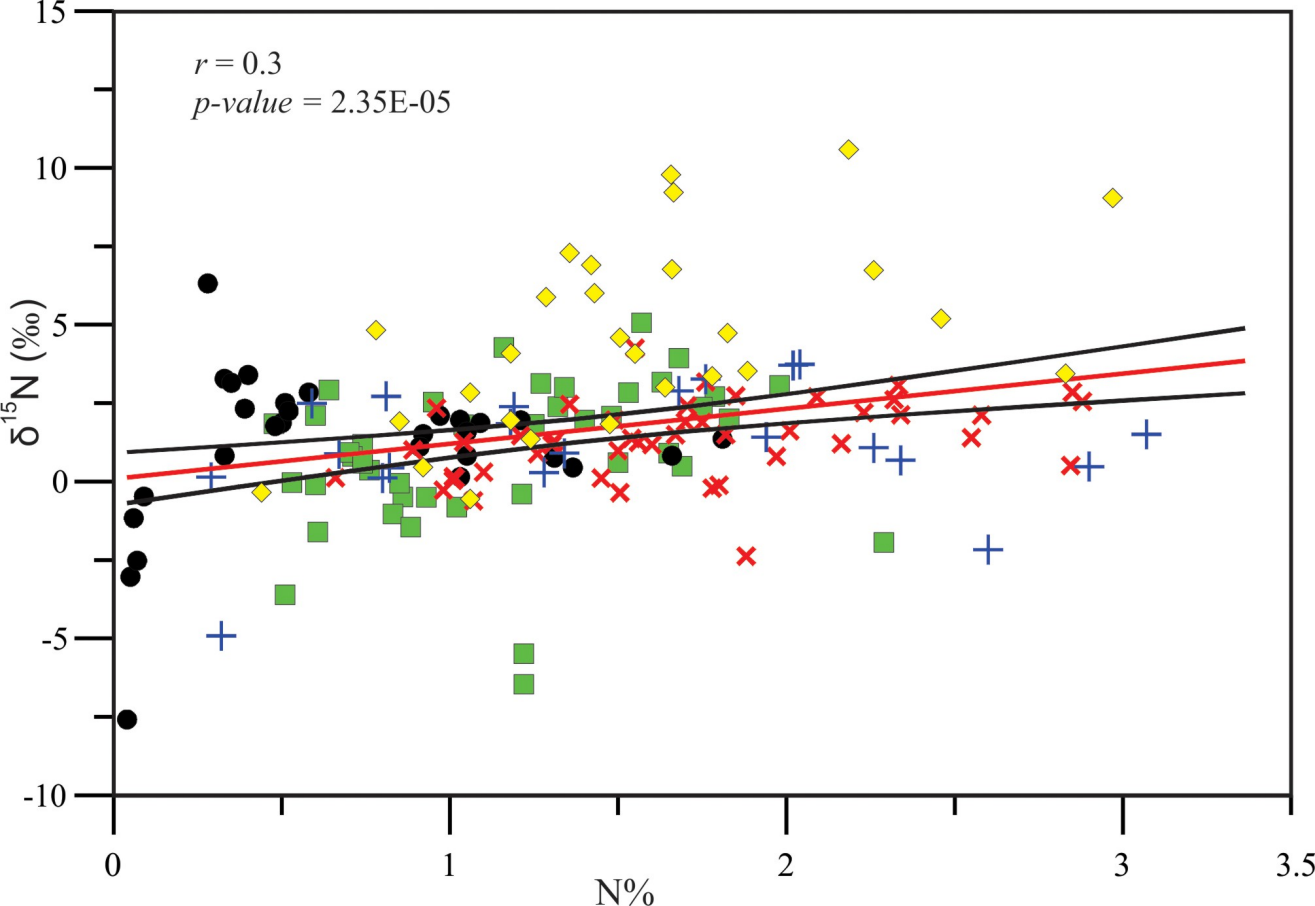
Relationship between N % and δ^15^N of the macrophytes (mangroves, halophytes, macroalgae and seagrasses) and sediments in/around the Al-Kharrar Lagoon. Red solid line indicates the fitted regression line between all macrophytes and sediments. The 95% confidence interval is indicated by black solid lines. For samples symbols see Figs 2–4.

The correlation between the elemental ratios (δ^13^C, δ^15^N and C/N) and C % and N % in the studied taxa and sediments indicated that as the C % in plant tissues increases, the N % increases (*r* = 0.47, *p-value* = 0.0001) (Fig 3). In this figure, the SOM are separated from the macrophytes showing the lowest C % and N % in/around the lagoon sediments. Also, as the C % in plant tissues increases, their δ^13^C values become more depleted (*r* = –0.13, *p-value* = 0.0001) (Fig 5). Whereas, as the N % increases, their δ^15^N values become more enriched (*r* = 0.3, *p-value* = 2.35E–05) (Fig 6). The relationship between the C/N ratio and δ^13^C shown in Fig 4 indicated the sediment samples have ratios of less than 10.

## Discussion

### The δ^13^C and δ^15^N of taxa and sediments

#### Mangroves and halophytes

The mangroves and halophytes live under subaerial conditions, comprising both aquatic and terrestrial settings and all provide essential roles sustaining their habitats [27]. In the KL, they showed more or less similar δ^13^C and δ^15^N values, separating them from the rest of the macrophytes in the lagoon (Fig 2). Their δ^13^C is the lightest among the studied macrophytes, possibly using a C3-photosynthetic pathway [4]. However, they are located under a dry, warm, tropical climate where availability of atmospheric CO_2_ for photosynthetic process is in favour of a C4-photosynthetic pathway and not C3-plants. The δ^13^C of C3-plants average –27 ‰ (range –32 ‰ to –22 ‰) and they mostly dominate temperate and cold regions where discrimination against ^13^C occurs [28–30]. Whereas, the δ^13^C of C4-plants averaged of –13 ‰ (range –16 ‰ to –10 ‰); they are dominant in warm, tropical regions where discrimination against ^13^C is nearly absent [31]. The only explanation for this is that the mangroves and halophytes could be adapted to this condition so preferentially uptake ^12^C during photosynthetic process. They live under stressful conditions, occupying supratidal and intertidal areas with hypersaline waters and have organs adapted to expel out excessive salts from their tissues [32]. Moreover, the lightest δ^13^C values in both mangroves and halophytes may be a result of their possessing a typical C3-photosynthetic pathway which could slightly be affected by droughts and the extremely high salinity in their substrates, which induce physiological processes such as photoinhibition and photooxidation. However, under environmental stressors, there are no earlier results indicating that mangroves and halophytes have changed towards a C4-photosynthetic pathway. In this study, only halophyte plants *Atriplex* sp. and *Suaeda vermiculata* showed δ^13^C values (–14.31 ‰) similar to plants of C4-photosynthetic pathway. They were found around the lagoon living on elevated sandy beaches, as well as in association with the other halophytes.

A notable feature is that the δ^13^C in the aerial roots (pneumatophores) of mangroves was slightly heavier (–25.28 ‰) than that of their leaves (–27.07 ‰) by 1.8 ‰. This is probably due to the fact that leaves use photosynthesis to build plant’s organic matter (OM), but roots are not, dependant on a supply from leaves to synthesize their OM and thus more fractionation may occur leading to enrichment of ^13^C in the roots. These results are consistent with a similar pattern observed in a greenhouse-based simulated mangrove *Aegiceras corniculatum* wetland system, where roots were heavier than leaves due to translocation of assimilated ^13^C-enriched carbohydrates to the roots [8, 33].

The δ^15^N of plants in the KL had a mean of 1.67±0.2‰, denoting to a predominance of the atmospheric N_2_ fixation by diazotrophic bacteria (δ^15^N = 0.0‰) as the main source of N, compared with the δ^15^N value for marine DIN of value around 5‰ [4, 9, 12, 26, 34]. It is also reported that a δ^15^N near 0.0 indicates a prevalence of atmospheric nitrogen to plant root systems [4]. The low value of δ^15^N in plants from the KL reflects their contribution as the first level components in the lagoonal ecosystem food web. They get their N from inorganic forms such as NO_3_^‒^ and NH_4_^+^, immediately after bacterial (mostly blue-green algae) N_2_-fixation and decomposition. This could lead to depletion of the δ^15^N of plant tissues [34], if this ^14^N is abundant and directly absorbed before modification by biogeochemical processes. These natural δ^15^N signatures of macrophytes in the KL could be used as a base-line for monitoring human-impacted coastal lagoons having a similar geographic setting. Another study was carried on the δ^15^N in seagrasses and macroalgae and recorded that values > 6 ‰ could reflect a significant influx of urban sewage and livestock effluent into the system [35]. However, high δ^15^N values could be also related to natural processes removing ^14^N from the environment, leaving the pool enriched in ^15^N [12]. To ease this contradiction, it is suggested to inspect whether the environment is connected to an anthropogenic source or not before deciding whether the highest δ^15^N values are related to natural or anthropogenic processes which may alter the isotopic signals of all associated habitats. The halophytes (saltmarshes) is the only plants in this study that showed the heaviest δ^15^N values (6–10.58‰) which are attributed to natural processes (discussed below) because the study area is far from any source of human contamination and the δ^15^N of most plants varied around 1.67‰.

In this study, halophytes and mangroves showed the most variable and heaviest δ^15^N values. This observed high δ^15^N and variability in the tissues of these plants could possibly be due to processes such as denitrification and ammonia evaporation occurring at their roots and substrates, leaving the pools enriched in ^15^N [36] since their roots grow in organic-rich sediments and far from urban activities [15, 21]. However, the aerial roots of some mangrove samples showed the most depleted δ^15^N value of –6.5 ‰. We noticed that the δ^15^N of halophytes collected from raised, aerated sandy substrates were lower (~ 0.0 ‰) than those collected from the supratidal area covered by algal mats and sabkha. It is possible that plants living in raised sandy substrates and under warm, dry climate may feed mostly on ^14^N-enriched NO_3_^–^ from rainfall, mostly during winter with δ^15^N between –5 to 0.0 ‰ [37] and denitrification could be very limited. However, denitrification may be abundant around the plant roots growing under the algal mats, leading to high δ^15^N values in living halophytes. It is reported that in natural ecosystems, denitrification and ammonia evaporation are the two main processes that increase the δ^15^N value [36]. The interannual variability of seagrasses δ^15^N from south Florida (USA) was studied and indicated that anomalous δ^15^N values are due to temporal changes in the isotopic composition of the DIN source induced by biogeochemical processes like N fixation, ammonification and denitrification [22].

#### Macroalgae and seagrasses

The δ^13^C of macroalgae A is more depleted (–15.41 ‰) than those of the macroalgae B which yielded the heaviest δ^13^C values (–7.41 ‰) similar to those of the seagrasses. These heavier values in the macroalgae and seagrasses may suggest that they are using a C4-photosynthetic pathway due to living in harsh environment with warm and hypersaline waters, so DIC (CO_2_) could be less available and consequently discrimination against ^13^C may be limited, leading to more enriched δ^13^C values [4]. In addition, they may use HCO_3_^–^ as a source of CO_2_ for photosynthesis, instead of dissolved CO_2_ which at isotopic equilibrium has a δ^13^C value identical to the atmospheric CO_2_ of –8 ‰, whereas HCO_3_^–^ is 7–9 ‰ heavier than DIC in a temperature ranging from 0 to 30°C [38]. Consequently, differential use of bicarbonate and dissolved CO_2_ could determine δ^13^C values of aquatic plants. Longinelli et al. [38] also reported that rapid carbon assimilation due to high light availability causes cell disequilibria which results in the preferential uptake of the heavier isotope. Overall, warm waters with high pH and light availability in the KL could force the macroalgae and seagrasses to derive much of their photosynthetic C from HCO_3_^–^ which has δ^13^C around 0.0 ‰ [26, 39, 40].

The δ^13^C in seagrass rhizomes was slightly heavier (similar to mangroves) than that of their leaves by 0.9 ‰. The reason for this may be as discussed above in the mangrove section due to translocation of carbon from leaves to roots. There is no notable change in the δ^13^C of the studied plants along the north-south salinity/temperature gradient within the KL. Also, the δ^13^C values of the studied plants from open seawater stations were similar to the ones collected from inside the lagoon.

The δ^15^N of macroalgae (1.5 ‰) and seagrasses (1.7 ‰) overlapped with an average of 2 ‰. The similarity between the δ^15^N of macroalgae and seagrasses could suggest that they get their N from the same source such as N_2_-fixed nitrogen which is directly absorbed by plants without much fractionation [41]. The macroalgae get their nutrients directly from the surrounding water via their tissues. Whereas, the seagrasses get their nutrients from the roots and rhizome. Under some circumstances they may get their nitrogen (DIN) from the water column instead of the interstitial pore water of sediments, especially when the DIN is more abundant in the water around the seagrasses, leading to a similar and heavier δ^15^N as in the macroalgae. Whereas in water with low nutrients the seagrasses depend on the DIN of sediments, leading to more depleted δ^15^N due to dominance of nitrification, a process dependent on degree of consumption and other processes competing for the same NH_4_^+^ substrates [42].

A notable feature is that the δ^15^N in seagrass rhizomes was slightly lighter (-0.70 ‰) than that of their leaves (1.71‰). The same was noticed in the mangroves where their aerial roots (pneumatophores) showed lighter δ^15^N (0.11 ‰) than that of their leaves (1.8 ‰). This is probably due to the roots acting as a conduits for fluids and bacterial N_2_-fixed nitrogen, so concentration and recycling of N in roots are probably not prolonged and do not experience much fractionation. This may lead to the occurrence of depleted δ^15^N in the roots. Whereas, leaves are the factory of organic matter (carbohydrates) in plants so fluids and nitrogen get fractionated and re-cycled, leading to more enriched δ^15^N in the leaves than in the roots. It is also possible that the occurrence of plenty of N_2_-fixaing bacteria attached to the roots of seagrasses (and mangroves) could directly provide these plants with nitrogen that could lead to more depleted δ^15^N values. The δ^15^N values of the rhizomes and leaves of seagrasses in the Arabian Gulf also showed similar patterns that as the δ^15^N of leaves increased, the rhizomes became depleted [23]. However, older seagrass leaves showed depleted δ^15^N and lower concentrations of N and C than younger parts of the leaves, suggesting more attention during sampling should be taken when studying nutrient flow in a food-web using seagrass leaves [6].

#### Sponges

In this study, the sponges had δ^13^C values of –10.72 ‰, centred between those of macroalgae and seagrasses which may mostly represent their diets, especially after decaying into fine particulates, probably with lesser quantities of bacteria and marine microalgae in the KL. This is because the lagoon is not connected to riverine organic carbon sources. In a lagoon from the central-east Gulf of California, the study of C and N isotopes of many organisms indicated that the suspended particulate organic matter and phytoplankton are the main organic source for food webs [43]. Sponges are filter feeders and get their food from particulate organic matter derived from the surrounding decayed macrophytes and coastal marine microalgae, so their tissue δ^13^C should approximate the δ^13^C of their diet, once a small enrichment of +0.5 ‰ to 1 ‰ is considered [12, 44]. It is mentioned that the δ^13^C values of the macrophytes did not change during decomposition, whereas the δ^15^N was subjected to large changes due to microbial activity [45]. This finding is supported with their δ^15^N values of 0.80±0.5 ‰ which overlapped with those of the macroalgae and seagrasses. This may also suggest that atmospheric N_2_-fixed nitrogen is dominant source in the KL and fractionation during the transfer of particulate organic nitrogen (PON) from the primary producers to sponges is nearly minimal.

#### Sediments

The δ^13^C of substrate sediment organic matter (SOM) underneath the studied macrophytes overlaps significantly with those of the macroalgae and to a lesser extent with the seagrasses and mangroves. This may suggest that the KL is at present a macroalgae-dominated basin which could be the most influencing parameters on the δ^13^C of the substrate sediments, a condition that could have been induced by the present warming. It is also noted that the δ^13^C of sediments collected from mangrove beds was more depleted than those collected from mud flats with vegetation of seagrasses and macroalgae, see the supporting information (S1 Table). In a similar study along the eastern Red Sea coast, the seagrass leaves and macroalgae blades were the major contributors to the organic matter accumulation in seagrass sediments, while mangrove leaves were the major contributors in mangrove sediments [18]. The δ^13^C of mangroves and halophytes are separated from those of macroalgae, seagrasses and substrate sediments, indicating that their contribution to the present KL bottom sediment is overwhelmed by those of macroalgae and seagrasses. On the other hand, the δ^13^C of SOM in the KL has a value of –15.52 ‰ centred between all the studied macrophytes. So, the δ^13^C of SOM could be an integration of all plant-types [46] which after death and decay they contribute to the composition of the SOM in the KL. The δ^13^C in sediments is a reflection of the dominant decayed-plants and marine coastal algae [36]. Therefore, the separation of subaerial mangroves and halophytes growing in supratidal-intertidal settings from the aquatic seagrasses and macroalgae based on their δ^13^C values could be a useful indicator to differentiate between marine-influenced and terrestrial-influenced sediments when interpreting paleo-sea level or paleoclimatic changes in coastal marine settings using the Holocene sediment archive. It is possible that during Holocene aridity periods (cold climate and lowering of sea level), the δ^13^C in sediments could be more depleted due to the dominance of mangroves and terrestrial plants, whereas during periods of warming and sea-level rise, the sediments could be more enriched due to the dominance of seagrasses and macroalgae. The findings that the δ^13^C of SOM is sourced from living plants, is also testified by the δ^15^N of the substrate sediments that showed values of 1.11 ‰ similar to that of the macroalgae, but with a high variability ranging from –7.59 to 6.32 ‰. There is no a notable change in the δ^15^N of the studied plants in the lagoon along a north-south gradient where salinity and temperature increase southwards. Also, the δ^15^N of the studied plants from open seawater stations were similar to those in the lagoon.

### C/N ratio, C % and N % in taxa and sediments

The C/N ratios of the macrophytes and sponges were high, but in their substrate sediments “end member” they were very low. This may be due to a considerable part of the produced organic matter from mangrove litter, seagrass leaves, dead macroalgae and halophytes in the KL do not stay inside the lagoon, but rather they get carried out to the open Red Sea waters by help of the dense, hypersaline waters of the lagoon [20, 21]. Another part of the produced organic matter from the primary producers in the lagoon could be blown into land by north-westerly wind during low tides and after accumulation and drying at shoreline. In the C/N ratio vs δ^13^C plot, most of sediment samples have ratios less than 10, indicating a greater contribution of the aquatic/lagoonal primary producers such as macroalgae and seagrasses to the SOM than that of the subaerial/terrestrial primary producers mangroves and halophytes. This estimate is based on the C/N ratio assigned by [16] and [17] who demonstrated that values below 10 indicate a marine origin and values of approximately10 indicate components of both marine and terrestrial origins in the sediment. It is also used as a useful proxy to differentiate between terrestrial and marine sources in ecosystems and to estimate the terrestrial contribution to the energy and nutrient supply to marine coastal systems [14, 17].

There is a tendency that as the C % in the tissues of the studied macrophytes in the KL increases, their δ^13^C values become more depleted, whereas as the N % increases their δ^15^N values become more enriched. Also, as C % increases in the studied plants, N % increases. The SOM showed the lowest C and N concentrations in/around the lagoon, so they are well separated from those of the macrophytes. The entrapment of low C and N in the sediments of KL is likely due to connection of the lagoon with the oligotrophic Red Sea basin and a high demand for nutrients by living fauna and flora in the lagoon which has no connection with a riverine input. Also as discussed above, removal of the accumulated organic materials by winds during low tides and during water circulation may be responsible for the occurrence of low C and N. A rapid turnover of the lagoon waters may allow a little settlement of organic matter in/on the lagoon sediments. This is because dense hypersaline subsurface waters, with high carrying capacity for detached organic/particulate materials, exit the lagoon into the open Red Sea waters and get replaced by clean, oligotrophic surface waters. These results are also consistent with that recorded by [18] who suggested that low concentrations of organic carbon in the Red Sea blue carbon habitats is partially attributable to the absence of rivers around the Red Sea, thereby limiting the supply of allochthonous C to these sediments. They also mentioned that organic carbon and N sediment stocks were closely coupled, reflecting the compositions of overlying vegetation and availability of N, limiting the sediment organic carbon reservoirs.

## Conclusions

The carbon stable isotopes (δ^13^C) of macrophytes in the Al-Kharrar Lagoon divided them into subaqueous/terrestrial and aqueous groups. The subaqueous/terrestrial plants (mangroves and halophytes) showed the most depleted δ^13^C values (~ –28 ‰), suggesting their use of C3-photosynthetic pathways, except for *Atriplex* sp. and *Suaeda vermiculata* (halophytes) which showed δ^13^C values of –14.31 ‰ similar to C4-plants. The aqueous plant macroalgae A (mostly red and brown) had δ^13^C values of –15.41 ‰, whereas macroalgae B (with green coloration) and seagrasses showed the most enriched δ^13^C values of –7.41 ‰, suggesting uptake of HCO_3_^–^ as a source of CO_2_ during photosynthesis. These divisions could be a useful indicator to differentiate between marine-influenced and terrestrial-influenced sediments when interpreting paleo-sea level or paleoclimatic changes from coastal marine archives. The δ^15^N of macrophytes, sponges and sediments in the KL are more or less the same with a mean value of 1.68 ‰, indicating that the main source of N in the KL is directly from atmospheric N_2_ fixation. However, denitrification may lead to high δ^15^N, up to 10.6 ‰ in the halophytes living in algal mats and sabkha substrates, whereas those living on raised, sandy beaches showed more depleted δ^15^N of 0.0 ‰, due possibly to their dependence on ^14^N-enriched NO_3_^‒^ from rainfalls. The aerial roots of some mangrove samples showed the most depleted δ^15^N of –5 ‰. The results of δ^13^C and δ^15^N could not differentiate between the macrophytes living in the lagoon and those living in the adjacent Red Sea waters. Also, they did not show any trend matching the environmental gradient inside the lagoon. The macrophytes in the KL showed higher values of C %, N %, and C/N ratio than their end member ‘sediments’. This is because the occurrence of rapid outflow of hypersaline subsurface waters with high macrophyte carrying capacity combined with their removal from shoreline by north-westerly winds. The C/N ratio of sediments was less than 10, indicating that terrestrial influx via rivers is very low. Since this lagoon is not connected to a source of pollution, the δ^15^N signals of its habitats and sediments could be used as a natural background when assessing human-impacted lagoons having similar geographic settings.

## Supporting information

S1 Table Station number list, coordinates and the values of C%, N%, C/N ratio, δ13C and δ15N in the tissues of taxa and sediments in Al-Kharrar Lagoon, collected October 2020. This table (XLSX) can be accessed in the SEANOE repository: https://doi.org/10.17882/98765.
(XLSX)
